# New Predictors of Early and Late Outcomes after Primary Percutaneous Coronary Intervention in Patients with ST-Segment Elevation Myocardial Infarction and Unprotected Left Main Coronary Artery Culprit Lesion

**DOI:** 10.1155/2019/8238972

**Published:** 2019-03-18

**Authors:** Cãlin Homorodean, Adrian Corneliu Iancu, Daniel Leucuţa, Şerban Bãlãnescu, Ioana Mihaela Dregoesc, Mihai Spînu, Mihai Ober, Dan Tãtaru, Maria Olinic, Dan Bindea, Dan Olinic

**Affiliations:** ^1^“Iuliu Hatieganu” University of Medicine and Pharmacy, Cluj-Napoca, Romania; ^2^Emergency County Hospital, Cluj-Napoca, Romania; ^3^Niculae Stãncioiu” Heart Institute, Cluj-Napoca, Romania; ^4^“Carol Davila” University of Medicine and Pharmacy, Bucharest, Romania

## Abstract

**Objectives:**

The study evaluated the correlation between baseline SYNTAX Score, Residual SYNTAX Score, and SYNTAX Revascularization Index and long-term outcomes in ST-elevation myocardial infarction (STEMI) patients with primary percutaneous coronary intervention (PCI) on an unprotected left main coronary artery lesion (UPLMCA).

**Background:**

Previous studies on primary PCI in UPLMCA have identified cardiogenic shock, TIMI 0/1 flow, and cardiac arrest, as prognostic factors of an unfavourable outcome, but the complexity of coronary artery disease and the extent of revascularization have not been thoroughly investigated in these high-risk patients.

**Methods:**

30-day, 1-year, and long-term outcomes were analyzed in a cohort of retrospectively selected, 81 consecutive patients with STEMI, and primary PCI on UPLMCA.

**Results:**

Cardiogenic shock (p=0.001), age (p=0.008), baseline SYNTAX Score II (p=0.006), and SYNTAX Revascularization Index (p=0.046) were independent mortality predictors at one-year follow-up. Besides cardiogenic shock (HR 3.28, p<0.001), TIMI 0/1 flow (HR 2.17, p=0.021) and age (HR 1.03, p=0.006), baseline SYNTAX Score II (HR 1.06, p=0.006), residual SYNTAX Score (HR 1.03, p=0.041), and SYNTAX Revascularization Index (HR 0.9, p=0.011) were independent predictors of mortality at three years of follow-up. In patients with TIMI 0/1 flow, the presence of Rentrop collaterals was an independent predictor for long-term survival (HR 0.24; p=0.049).

**Conclusions:**

In this study, the complexity of coronary artery disease and the extent of revascularization represent independent mortality predictors at long-term follow-up.

## 1. Introduction

ST-segment elevation myocardial infarction (STEMI) with unprotected left main coronary artery (UPLMCA) culprit lesion accounts for 0.8-1.2% of all primary percutaneous coronary interventions (PCI) [1–3].

Retrospective studies have identified cardiogenic shock, TIMI 0/1 flow, and cardiac arrest as the main prognostic factors of an unfavorable outcome [1, 3–6]. In these studies, due to limitations of retrospective data collection, one-year mortality rates vary between 11 and 69% [1, 3–15]. Several registries [16, 17] have demonstrated better outcomes with coronary artery bypass grafting (CABG) in patients with acute coronary syndromes and UPLMCA culprit lesions. The higher frequency of complete revascularization with CABG, as compared to PCI, may well represent a predictor of long-term survival. Until now, the initial complexity of coronary artery disease (CAD) and the extent of revascularization have not been thoroughly investigated as outcome predictors in this group of patients.

The aim of this study was to evaluate the correlation between baseline SYNTAX Score, Residual SYNTAX Score, SYNTAX Revascularization Index, and long-term survival in the setting of STEMI with UPLMCA culprit lesion.

## 2. Materials and Methods

**Figure 1:**
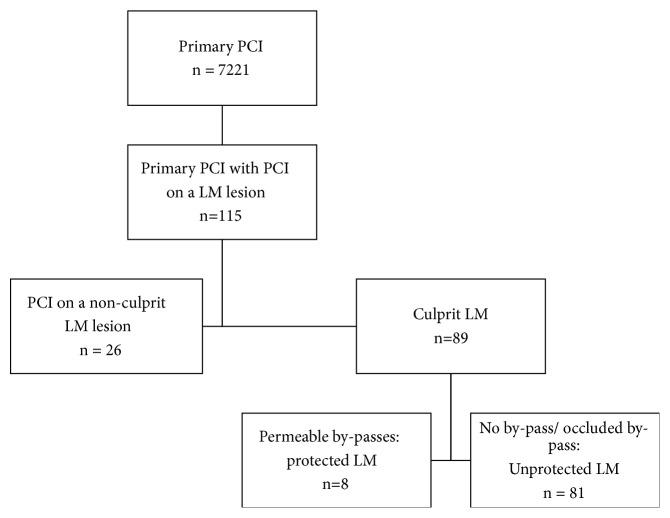
Flow chart. LM=left main; PCI= percutaneous coronary intervention.

This was a retrospective observational cohort study. Patients with STEMI and primary PCI for UPLMCA culprit lesion were identified in the registries of two centers in Cluj-Napoca, Romania (Figure 1). The inclusion period was between January 2010 and March 2017.

The inclusion criteria were ongoing ischemic chest pain with a duration of more than 30 min, accompanied by ST-segment elevation of at least 0.2mV in two contiguous electrocardiographic (ECG) leads, left main STEMI equivalent ECG changes [11, 18], new left bundle branch block, and/or cardiogenic shock.

Coronary flow was graded according to the TIMI classification system. Collateral flow was evaluated using Rentrop criteria [19].

Left main was considered “unprotected” in the absence of any patent left coronary artery bypass grafts.

UPLMCA was considered the culprit vessel in case of a more than 90% stenosis or in case of an angiographic complicated lesion: dissection, thrombus, plaque rupture, or TIMI 0-2 flow [6].

Baseline SYNTAX Score I, baseline SYNTAX Score II, residual SYNTAX Score, and EuroScore II were calculated for each patient by two independent senior interventional cardiologists. SYNTAX Score Revascularization Index (SRI) represents the proportion of CAD burden treated by PCI [20]. It was calculated using the formula: SRI=(1–[rSS/bSS])x100.

Technical success was defined as less than 30% residual stenosis in the presence of TIMI 3 flow [5]. Provisional stenting was the preferred strategy, due to presumed less devices manipulation and distal embolization. The procedures were performed by eight senior operators, each of them performing more than 250 coronary interventions annually.

All patients were monitored in acute coronary care units and received standard medical treatment, according to the current practice guidelines.

Information regarding outcomes was obtained from hospital or primary care physician records, telephone interviews, questionnaires sent by mail, or domicile visits. Data on the vital status was available in the electronic records of the national insurance company. Follow-up ended on 31 December 2017.

Major adverse cardiac events (MACE) included death, nonfatal myocardial infarction, and target vessel revascularization (TVR). All-cause mortality was reported according to Academic Research Consortium recommendations [21].

### 2.1. Statistical Analysis

Continuous variables were presented as mean ± standard deviation, or median and interquartile range. Categorical variables were presented as numbers or proportions.

To assess the relation between variables of interest and survival, Cox proportional hazard regressions were used. Unadjusted models were first build, followed by models that included the variables of interest. These were adjusted for the presence of shock and TIMI flow 2/3 vs. 0/1. The proportional hazard assumption was assessed graphically with the Schoenfeld residuals and with a formal statistical test. Martingale residuals were used to identify the functional form for the influence of variables. The log-linearity assumption was checked for continuous variables with penalized smoothing splines graphically and formally with a statistical test. The presence of multicollinearity was checked for all adjusted regressions. Adjusted survival curves were plotted and predicted with a multivariate Cox model.

Logistic regressions were used to similarly model the relation between variables of interest and death. The odds ratio along with 95% log likelihood confidence intervals and p-values were computed for each regression. The goodness-of-fit, the presence of multicollinearity, and misspecification were checked for each model. Area under the receiver operator characteristic curve (AUC) for logistic regression was presented along with 95% confidence intervals. Optimal cut-off points were identified using the Youden index.

A two-sided p-value < 0.05 was considered statistically significant.

All analyses were performed with R environment for statistical computing and graphics (R Core Team. R: A Language and Environment for Statistical Computing [Internet], Vienna, Austria, 2017).

## 3. Results

### 3.1. Baseline Clinical and ECG Data

The sample consisted of 81 PCI-treated patients with STEMI and UPLMCA culprit lesion. Baseline clinical characteristics and ECG changes are presented in Table 1. Electronic data on mortality was available in all patients. Medical records were obtained in 90% of the patients.

Median total ischemic time was 360 min (IQR 142.5 - 600). On admission, cardiogenic shock was present in 49.4% of patients, while 34.5% had resuscitated cardiac arrest. Median EuroSCORE II was 12.2 (IQR 3.88 - 28.6).

### 3.2. Angiographic and Procedural Characteristics

Angiographic and procedural characteristics are summarized in Table 2.

TIMI 0/1 flow was present in 23.4% of the patients. Of these, 68.4% were in cardiogenic shock. In the setting of TIMI 0/1 flow, right coronary artery Rentrop collaterals were identified in 52.6% of cases. Left main bifurcation disease was observed in 51.8% and multivessel CAD in 69.2% of patients. Median baseline SYNTAX Score I was 28 (IQR 18 - 34) and mean baseline SYNTAX Score II was 43.1±15.76.

Drug-eluting stents (DES) were used in 40.7% of cases.

In 61.7% of cases, the stent was placed across the origin of the left circumflex artery. Kissing balloon technique was performed in 26% of these patients and it was followed by proximal optimization in 83% of them. Overall, proximal optimization was performed in 50% of cases when the stent was placed across left circumflex origin.

Two-stent techniques were applied in 8.6% of patients with bifurcation lesions.

Technical success was 92.6%. Median residual SYNTAX Score was 2 (IQR 0 - 11), while median SRI was 89.4 % (IQR 64.3 - 100).

### 3.3. 30 Days Follow-Up

Clinical outcomes at 30 days, one-year, and long-term follow-up are summarized in Table 3.

Mortality in the catheterization laboratory was 6.2%, while in-hospital mortality reached 30.8%.

The overall 30-day mortality rate was 35.8%. In patients with cardiogenic shock it reached 60%, while in stable patients it was 4.9 times lower (p<0.001).

Death occurred in 63.15% of patients with TIMI 0/1 flow at 30-day follow-up, and it was 2.4 times lower in case of TIMI 2/3 flow (p=0.004).

Among patients with TIMI 0/1 flow, mortality was 100% in those with noncollateralized left coronary artery and 30% in those with collaterals.

**Figure 2:**
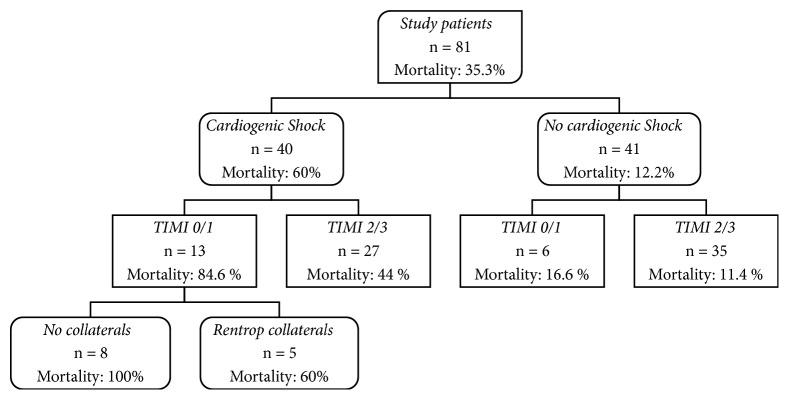
Thirty-day mortality in different patient subgroups—patients are grouped by cardiogenic shock, TIMI flow, and collaterals. TIMI = Thrombolysis in Myocardial Infarction.

Mortality at 30-day follow-up was 84.6% in the group of patients with both TIMI 0/1 flow and cardiogenic shock. In the same group, mortality was 60% when collaterals were present. In the absence of cardiogenic shock, 30-day mortality was 16.6% when TIMI flow was 0/1 and 11.4% when TIMI flow was 2/3 (Figure 2).

In-hospital mortality was 64.2% in patients with prerevascularization resuscitated cardiac arrest and 20.7% in those without.

Cardiogenic shock (p<0.001), TIMI 0/1 flow (p=0.006), resuscitated cardiac arrest (p<0.001), low ejection fraction (p=0.018), Euro SCORE II (p=0.01), baseline SYNTAX Score I (p=0.023), baseline SYNTAX Score II (p=0.01), residual SYNTAX Score (p=0.01), and SRI (p=0.008) were identified as predictors for mortality by univariate logistic regression analysis (Supporting Table 1). Using multivariate logistic regression analysis, cardiogenic shock (p<0.001) and TIMI 0/1 flow (p=0.038) emerged as independent mortality predictors (Table 4).

### 3.4. One-Year Follow-Up

At one-year follow-up, seven additional deaths occurred and led to an all-cause mortality rate of 44.4%. Only three of these had cardiac causes. The rate of one-year MACE was 49.38%. Three cases of TVR that were due to intra-bare-metal stent restenosis and one nonfatal myocardial infarction were recorded.

ROC curve analysis demonstrated a significant association (p=0.022 and 0.020) between both residual SYNTAX Score and SRI and one-year all-cause mortality. A SRI cut-off of 84% had the best prognostic accuracy for risk prediction of all-cause mortality (AUC of 64.2, 95% CI: 52.4-76.05), while for residual SYNTAX Score the optimal cut-off value was 7.5 (AUC of 64.38, 95%CI: 52.58-76.19). A comparison between the predictive capabilities of residual SYNTAX Score and SRI is depicted in Table 5.

Using univariate logistic regression analysis, cardiogenic shock (p<0.001), initial TIMI 0/1 flow (p=0.02), resuscitated cardiac arrest (p=0.003), Euro SCORE II (p=0.005), baseline SYNTAX Score I (p=0.02), baseline SYNTAX Score II (p<0.001), residual SYNTAX Score (p=0.012), and SRI (p=0.009) remained predictors of mortality at one-year follow-up. Other predictors were age (p=0.03) and bare-metal stent implantation (p=0.046) (Supporting Table I).

Multivariate analysis identified cardiogenic shock (p=0.001), age (p=0.008), baseline SYNTAX Score II (p=0.006), and SRI (p=0.046) as mortality predictors at 1-year follow-up (Table 4).

### 3.5. Long-Term Follow-Up

Among the 30-day survivors, mean follow-up was 36 months (3 years). Between one-year and final follow-up, eight other patients died, two of them of cardiac causes. All-cause mortality reached 54.3%.

**Figure 3:**
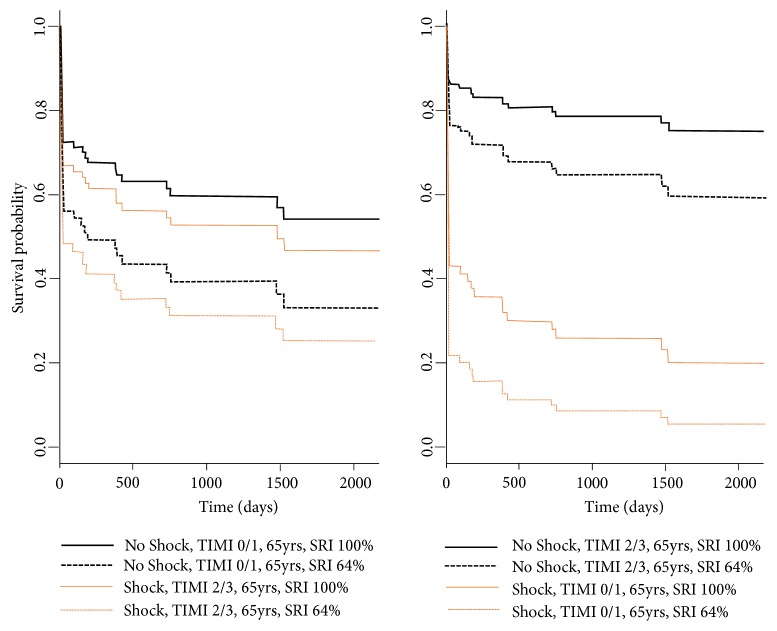
Predicted survival curves: the survival curves are predicted with a multivariate Cox model for the first (64%) and the third (100%) quartiles of SYNTAX Revascularization Index in different scenarios: with and without shock, TIMI 0/1 or 2/3, and adjusted for the subjects mean age (65 years). TIMI =Thrombolysis in Myocardial Infarction; yrs = years.

Using univariate Cox-regression analysis (Table 6) cardiogenic shock on admission (p<0.001), TIMI 0/1 flow (p=0.012), cardiac arrest before PCI (p=0.005), age (p=0.025), SYNTAX Score I (p=0.002) and II (p<0.001), residual SYNTAX Score (p<0.001) and SRI (p<0.001), and bare-metal stent usage (p=0.03) were identified as long-term survival predictors. On multivariate analysis (Table 6) cardiogenic shock (p<0.001), TIMI 0/1 flow (p=0.021), age (p=0.006), SRI (p=0.011), and residual SYNTAX Score (p=0.041) remained independent predictors for mortality. Predicted, adjusted survival curves for SRI are shown in Figure 3, in different scenarios: with and without shock, TIMI 0/1, or 2/3.

In patients with TIMI 0/1 flow, the presence of right coronary artery collaterals was an independent predictor of long-term survival (HR: 0.24; 95%CI: 0.06-0.99; p=0.049) when adjusted for the presence of cardiogenic shock and age.

## 4. Discussion

The present study evaluated a group of patients with acute STEMI and primary PCI on a culprit UPLMCA.

All-cause mortality at 30 days reached 35.8%, being comparable to previous studies [1–8, 10, 11, 13, 14]. The majority of deaths occurred in the first 30 days from the index event, and seven additional deaths were recorded between 30-day and one-year follow-up. The results were similar in other studies [10].

At short- and long-term follow-up, two classes of independent predictors of mortality were identified.

The first category included parameters which reveal the complexity of the initial (baseline SYNTAX Score I and II) and remaining CAD (residual SYNTAX Score), as well as parameters which characterize the extent of revascularization (residual SYNTAX Score and SRI) [20, 22–24]. Baseline SYNTAX Score II emerged as an independent predictor of mortality at 1-year follow-up.

The residual SYNTAX Score and SRI values showed that most frequently complete revascularization was not achieved. In fact, SRI was originally introduced to quantify the level of “reasonable incomplete revascularization” [20]. SRI and residual SYNTAX Score emerged as independent predictors of mortality at 3-year follow-up.

Both SRI and residual SYNTAX Score had a moderate capacity in predicting mortality after 1 year with an AUC value of 0.64. The curves and the derived optimal cut-off values are comparable to those derived from larger studies [20, 23]. A SRI value greater than 84% of the baseline SYNTAX Score predicted survival after 1 year. Actually, the importance of complete revascularization could explain why CABG outplayed PCI in previous studies [1, 4, 10, 17]. A selection bias was also present in these studies, since cardiogenic shock was an independent predictor of PCI treatment allocation [1, 4]. In the prospective CUSTOMIZE registry, a significantly larger proportion of patients in the CABG group underwent complete revascularization as compared to the ones in the PCI group [17]. Furthermore, PCI remained associated with a higher risk of MACE [4, 17]. However, a selection bias existed here too. The PCI group had higher risk patients since STEMI was significantly more frequently associated with percutaneous treatment [17].

Regarding residual SYNTAX Score, it has been shown to provide good accuracy in predicting future ischemic events in non-ST-elevation acute coronary syndromes [20], in primary PCI [24], and even in patients with stable left main disease [22].

To the best of our knowledge the present study was the first to identify SRI and residual SYNTAX Score as independent predictors for survival in PCI treated STEMI patients with UPLMCA culprit lesion.

A second category referred to mortality predictors linked to the initial presentation. Cardiogenic shock was the most important predictor of death together with TIMI 0/1 flow and contralateral collateralization. The results were consistent with previously published data [1, 3–8, 10, 11, 13]. The prevalence of shock (45-78%) determined the magnitude of mortality as it represented the most constant outcome predictor [1, 5, 6, 8, 10, 11]. In what concerns TIMI 0/1 flow [1, 8, 11] and the presence of cardiac arrest prior to revascularization [5, 6], they were less constantly proven to be negative outcome predictors.

The prevalence of TIMI 0/1 flow in the studied population was rather low (23.4%) when compared to the one previously reported [1, 5, 7]. Longer total ischemic time might have played a role in this result [1, 4–6]. Actually, this was described by Grundeken [1] as the “time paradox”, caused by survival bias. One can speculate that some critical patients with TIMI 0/1 flow in the absence of collateralization died before reaching any medical facility.

This study demonstrated that the presence of collaterals was an independent predictor of survival at long-term follow-up in patients with STEMI, UPLMCA culprit lesion, and TIMI 0/1 flow. Collaterals presence ranged between 10-15% [10] and 48% in studies with a more favorable outcome [1].

Age was also an independent mortality predictor at one-year follow-up. Chieffo et al. [25] have presented a similar result.

To summarize, both cardiogenic shock and TIMI flow were crucial parameters as they predicted mortality both on the short- and on the long-term. Age, lesion complexity, and the extent of revascularization were medium- and long-term mortality predictors, while the presence of residual lesions influenced mortality only on the long-term.

One important limitation arises from the inherent long inclusion period, a consequence of the low frequency of eligible cases. The most important change in practice that occurred during this time interval regarded the use of new generation DES. However, only in 2017 did the European practice guidelines introduce a class IA recommendation for the use of DES in the setting of STEMI [26]. The highest rate of BMS restenosis is encountered when a two-stent technique is used. In this study 8.6% of patients were treated with two stents and in all cases DES were used.

Other limitations are the result of its retrospective character, with all the selection biases it confers. The lack of systematic angiographic control represents a main issue since it might have led to MACE underestimation.

Previous studies demonstrated better outcomes with CABG as compared to PCI in patients with acute coronary syndromes and UPLMCA culprit lesion. The higher frequency of complete revascularization could explain why CABG had better outcomes as compared to PCI in this setting. The present study identified the extent of revascularization as an independent predictor for survival at long-term follow-up in patients with STEMI and primary PCI for an UPLMCA culprit lesion. The question whether complete revascularization should be targeted in this specific setting, irrespectively of the presence of cardiogenic shock, should be answered in a large, prospective, randomized trial.

## 5. Conclusion

Primary PCI in STEMI patients with UPLMCA is accompanied by a high 30-days mortality, which decreases during the following months. Baseline SYNTAX Score II, residual SYNTAX Score, and SRI represent independent mortality predictors. Actually, the severity of baseline coronary artery disease and the extent of revascularization independently affect survival at long-term follow-up. Cardiogenic shock, TIMI 0/1 flow, and lack of collaterals in the TIMI 0/1 flow subgroup were reconfirmed as independent predictors of an adverse outcome.

## Figures and Tables

**Table 1:** Baseline clinical and ECG characteristics. Values are mean ± standard deviation, median (interquartile range), or number (%). AMI = acute myocardial infarction, IABP = intra-aortic balloon pump, IQR = interquartile range, N = number, and PCI = percutaneous coronary intervention.

Baseline patient characteristics	N = 81
Age (years)	65 ± 13.6
Male	59 (72.8)
Diabetes mellitus	27 (33.3)
Hypertension	49 (60.5)
Smokers	27 (33.3)
Previous AMI	15 (18.5)
Previous PCI	6 (7.4)
Previous stroke	6 (7.4)
Cardiogenic shock	40 (49.4)
Cardiac arrest before PCI	28 (34.5)
Total ischemic time (min)	360 (142.5-600)
IABP	6 (7.4)

**Table 2:** Angiographic and procedural characteristics. Values are mean ± standard deviation, median (inter-quartile range), or number (%). BMS = Bare-Metal Stent, DES = Drug-Eluting Stent, LCA = left coronary artery, LM = Left Main, PCI = percutaneous coronary intervention, RCA = right coronary artery, and TIMI = Thrombolysis in Myocardial Infarction.

Angiographic characteristics	N = 81
Lesion Location	
Proximal LM	31 (38.3)
Mid LM	8 (9.9)
Distal LM	42 (51.8)
Pre-procedural TIMI Flow Grade	
TIMI 0	11 (13.6)
TIMI 1	8 (9.9)
TIMI 2	18 (22.2)
TIMI 3	44 (54.3)
Rentrop collaterals to LCA	10
Occluded RCA	16 (19.7)
Rentrop collaterals to RCA	11 (13.5)
Extent of coronary artery disease	
LM only	25 (30.8)
LM + 1vessel	19 (23.5)
LM + 2 vessels	20 (24.7)
LM + 3 vessels	17 (21)
LM stenosis	
70-89%	20 (24.7)
90-99%	49 (60.5)
100%	12 (14.8)
EuroScore II	12.2 (3.88-28.6)
SYNTAX Score I	28 (18-34)
Residual SYNTAX Score	2 (0-11)
SYNTAX Revascularization Index	89.4 (64.3-100)
SYNTAX Score II PCI	43.1±15.7
Procedural characteristics	
Bifurcation technique	56 (69.1)
Kissing stents	3 (3.7)
Provisional	49 (60.5)
V stenting	1 (1.2)
T stenting	2 (2.5)
Mini crush	1 (1.2)
BMS	48 (59.3)
DES	33 (40.7)
Post-PCI TIMI Flow	
0	0
1	5 (6.2)
2	5 (6.2)
3	71(87.6)
Technical success	76 (92.8)

**Table 3:** Outcomes at 30-day, 1-year, and 3-year follow-up. Values are number (%). MACE = major adverse cardiac events, MI = myocardial infarction, and TVR = target vessel revascularization.

Outcomes	N = 81
Catheterization Laboratory mortality	5 (6.2)
In-hospital mortality	25 (30.8)
30-days all-cause mortality	29 (35.8)
1-year outcomes	
All-cause mortality	36 (44.4)
TVR	3 (3.7)
Non-fatal MI	1(1.2)
MACE	40 (49.38)
Long term all-cause mortality (3 years)	44 (54.3)

**Table 4:** Multivariate logistic regression for the prediction of 30 days and 1-year all-cause mortality (adjusted for cardiogenic shock and TIMI flow). BMS = bare-metal stent, DES = drug-eluting stent, LM = Left Main, PCI = percutaneous coronary intervention, and TIMI = Thrombolysis in Myocardial Infarction.

	30 days			One year		
	OR	95% CI	p	OR	95% CI	p
LM TIMI flow 0/1	3.73	1.107-13.7	0.038	2.89	0.91-9.9	0.077
Cardiogenic shock before PCI	9.83	3.28-34.65	< 0.001	5.13	1.96-14.26	0.001
Age (years)	1.05	1.002-1.101	0.053	1.06	1.02-1.12	0.008
Total ischemic time (min)	1	0.99-1.003	0.966	1	0.99-1.002	0.98
Diabetes	1.94	0.61-6.36	0.261	1.21	0.42-3.49	0.71
Cardiac arrest before PCI	1.78	0.47-6.53	0.386	1.47	0.39-5.29	0.55
Left ventricular ejection fraction (<40%)	1.54	0.37–6.89	0.551	1.19	0.35–4.06	0.76
EuroSCORE II	1.001	0.97-1.035	0.941	1.02	0.98-1.05	0.30
Number of diseased vessels	1.05	0.62-1.78	0.849	1.405	0.88-2.30	0.16
SYNTAX Score	1.02	0.97-1.078	0.414	1.029	0.98-1.08	0.252
SYNTAX Revascularization Index	0.97	0.95-1	0.057	0.979	0.95-0.99	0.046
PCI SYNTAX II Score	1.03	0.99-1.07	0.163	1.059	1.02-1.11	0.006
Residual SYNTAX Score	1.05	0.99-1.11	0.107	1.053	1-1.12	0.069
DES vs BMS	0.74	0.23-2.38	0.617	0.46	0.15-1.3	0.148

**Table 5:** Predictive capabilities of residual SYNTAX Score and SYNTAX Revascularization Index on one-year all-cause mortality. AUC = area under the curve, rSS: residual SYNTAX Score, and SRI: SYNTAX Revascularization Index.

	AUC (95%CI)	Optimal cut-off	Sensitivity (%)	Specificity (%)
rSS	64.38 (52.58-76.19)	7.5	75.5	50
SRI	64.2 (52.4-76.05)	84	64	58

**Table 6:** Univariate and multivariate Cox survival analyses adjusted for cardiogenic shock, initial TIMI flow, and age. BMS = bare-metal stent, DES = drug-eluting stent, LM = Left Main, PCI = percutaneous coronary intervention, and TIMI = Thrombolysis in Myocardial Infarction.

	Univariate			Multivariate		
	HR unadjusted	95% CI	p	HR adjusted	95% CI	p
LM TIMI flow 0/1	2.27	1.20-4.34	0.012	2.17	1.13 – 4.16	0.021
Cardiogenic shock before PCI	3.09	1.64-5.80	<0.001	3.28	1.74 - 6.21	<0.001
Age (years)	1.02	1.003-1.05	0.025	1.03	1.01 - 1.06	0.006
Cardiac arrest before PCI	2.34	1.28-4.27	0.005	1.34	0.58-3.12	0.489
SYNTAX Score	1.04	1.016-1.07	0.002	1 01	0.97 - 1.04	0.587
SYNTAX Revascularization Index	0.97	0.96-0.98	<0.001	0.98	0.97 - 0.99	0.011
PCI SYNTAX II Score	1.04	1.02-1.06	<0.001	1.03	0.98 - 1.04	0,253
Residual SYNTAX Score	1.06	1.03-1.086	<0.001	1.03	1.001 - 1.06	0.041
DES vs BMS	0.45	0.22-0.92	0.03	0.58	0.28 - 1.19	0.139

## Data Availability

Data used to support the findings of this study are available from the corresponding author upon request.

## Supplementary Materials

Supplementary Materials Supporting Table 1: Univariate analysis for the prediction of the 30-day and one-year all-cause mortality; BMS = bare-metal stent, DES = drug-eluting stent, LM = Left Main, PCI = percutaneous coronary intervention, and TIMI = Thrombolysis in Myocardial Infarction.
