# Supplementary material for: New Predictors of Early and Late Outcomes after Primary Percutaneous Coronary Intervention in Patients with ST-Segment Elevation Myocardial Infarction and Unprotected Left Main Coronary Artery Culprit Lesion
Source: J Interv Cardiol. 2019 Mar 18;2019:8238972. doi: 10.1155/2019/8238972 (PMC6739789; doi:10.1155/2019/8238972)
Supplement: Supplementary Materials — Supporting Table 1: Univariate analysis for the prediction of the 30-day and one-year all-cause mortality; BMS = bare-metal stent, DES = drug-eluting stent, LM = Left Main, PCI = percutaneous coronary intervention, and TIMI = Thrombolysis in Myocardial Infarction. [file 8238972.f1.pdf]

Supporting Table 1: Univariate analysis for the prediction of the 30-days and one-year all-cause mortality

BMS = bare-metal stent, DES = drug-eluting stent, LM = Left Main, PCI = percutaneous coronary intervention, TIMI = Thrombolysis in Myocardial Infarction.

	30 days			One year		
	OR	95% CI	p	OR	95% CI	p
LM TIMI flow 0/1	4.54	1.56-14.08	0.006	3.67	11.76–1.27	0.02
Cardiogenic shock before PCI	10.8	3.73-36.87	< 0.001	5.75	2.25-15.70	< 0.001
Age (years)	1.02	0.98-1.062	0.198	1.04	1.005-1.08	0.03
Total ischemic time (min)	1	0.99-1.002	0.684	0.73	0.27-1.98	0.54
Diabetes	1.74	0.66-4.54	0.253	1	0.99-1.002	0.733
Cardiac arrest before PCI	6.87	2.55-19.83	< 0.001	4.47	1.72-12.39	0.003
Left ventricular ejection fraction (<40%)	4.24	1.39–15.87	0.018	2.76	1.02–8.06	0.051
EuroSCORE II	1.03	1.009-1.06	0.01	1.04	1.01-1.07	0.005
Number of diseased vessels	0.98	0.65-1.47	0.937	1.22	0.83-1.83	0.312
SYNTAX Score I	1.05	1.009-1.10	0.023	1.07	1.01-1.101	0.022
SYNTAX Revascularization Index	0.97	0.95-0.99	0.008	1.06	0.95-0.99	0.009
PCI SYNTAX II Score	1.04	1.01-1.08	0.01	0.37	1.03-1.11	< 0.001
Residual SYNTAX Score	1.06	1.02-1.12	0.01	1.05	1.02-1.13	0.012
DES vs BMS	0.53	0.19-1.4	0.21	0.97	0.14-0.96	0.046

For Peer Review
